# RAB39B-mediated trafficking of the GluA2-AMPAR subunit controls dendritic spine maturation and intellectual disability-related behaviour

**DOI:** 10.1038/s41380-021-01155-5

**Published:** 2021-05-25

**Authors:** Maria Lidia Mignogna, Stefano Musardo, Giulia Ranieri, Susanna Gelmini, Pedro Espinosa, Paolo Marra, Sara Belloli, Valentina Murtaj, Rosa Maria Moresco, Camilla Bellone, Patrizia D’Adamo

**Affiliations:** 1 Neuroscience Division, Unit of molecular genetics of intellectual disability, IRCCS San Raffaele Scientific Institute, Milan, Italy; 2 Department of Basic Neurosciences, University of Geneva, Geneva, Switzerland; 3 Preclinical Imaging Facility, Experimental Imaging Center (EIC), IRCCS San Raffaele Scientific Institute, Milan, Italy; 4 Institute of Bioimaging and Physiology, CNR, Segrate (MI), Italy; 5 Experimental Imaging Center (EIC), IRCCS San Raffaele Scientific Institute, Milan, Italy; 6 PhD Program in Neuroscience, University of Milano - Bicocca, Monza, Italy; 7 Medicine and Surgery Department, University of Milano - Bicocca, Monza (MB), Italy

**Keywords:** Neuroscience, Biological techniques

## Abstract

Mutations in the *RAB39B* gene cause X-linked intellectual disability (XLID), comorbid with autism spectrum disorders or early Parkinson’s disease. One of the functions of the neuronal small GTPase RAB39B is to drive GluA2/GluA3 α-amino-3-hydroxy-5-methyl-4-isoxazolepropionic acid receptor (AMPAR) maturation and trafficking, determining AMPAR subunit composition at glutamatergic postsynaptic neuronal terminals. Taking advantage of the *Rab39b* knockout murine model, we show that a lack of RAB39B affects neuronal dendritic spine refinement, prompting a more Ca^2+^-permeable and excitable synaptic network, which correlates with an immature spine arrangement and behavioural and cognitive alterations in adult mice. The persistence of immature circuits is triggered by increased hypermobility of the spine, which is restored by the Ca^2+^-permeable AMPAR antagonist NASPM. Together, these data confirm that RAB39B controls AMPAR trafficking, which in turn plays a pivotal role in neuronal dendritic spine remodelling and that targeting Ca^2+^-permeable AMPARs may highlight future pharmaceutical interventions for *RAB39B*-associated disease conditions.

## Introduction

Genetic alterations, either copy number gain, deletions or point mutations, of the X-linked *RAB39B* locus are associated with complex neuropathological clinical features, ranging from X-linked intellectual disability (XLID) in comorbidity with autism spectrum disorders, macrocephaly and seizures (OMIM: 300774) [1–4] or with early Parkinson’s disease (OMIM: 311510) [5–11]. RAB39B is a neuronal RAB GTPase that controls intracellular vesicular trafficking by switching from an active GTP-bound state to an inactive GDP-bound state [12]. We previously showed that RAB39B, in tandem with PICK1, drives GluA2/GluA3 α-amino-3-hydroxy-5-methyl-4-isoxazolepropionic acid receptor (AMPAR) trafficking throughout the secretory pathway to achieve AMPAR subunit insertion at the neuronal glutamatergic postsynaptic terminal. The downregulation of RAB39B in primary hippocampal neurons alters GluA2/GluA3 AMPAR trafficking from the endoplasmic reticulum (ER) to the Golgi apparatus, affecting AMPAR subunit composition as a final step and leading to increased surface expression of GluA2-lacking Ca^2+^-permeable AMPARs [13].

The subunit composition of AMPARs is highly dynamic and is modified during development, in cases of plasticity and by disease. The increase in surface expression of GluA2-lacking Ca^2+^-permeable AMPAR composition is mostly observed after plasticity-inducing neuronal activity [14, 15] or in young animals. Pathological conditions have been associated with immature synapses and cognitive impairment [16].

*RAB39B*, similar to many other XLID genes, plays a role in a common cellular pathway that controls glutamatergic synapses during development and network establishment [17, 18]. One of the most important cellular processes during brain development is spine formation. This event occurs during early postnatal development and is followed by a pruning phase during adolescence when redundant synaptic connections are removed and remodelled to create a mature state [19–21]. During the adult phase, a constant equilibrium between dendritic spine formation and elimination persists, leading to spine maintenance [22, 23]. However, the molecular mechanism underlying spine development and maturation is not totally defined.

Here, by using a *Rab39b* knockout (KO) murine model, we confirmed that the complete absence of RAB39B leads to increased surface expression of GluA2-lacking Ca^2+^-permeable AMPARs, which results in an immature spine state caused by unsuccessful neuronal dendritic spine refinement associated with cognitive deficits. Moreover, the persistence of immature spines and aberrant circuits could be triggered by an increase in the hyperdynamic features of spines, often observed at the early developmental neuronal stage.

Last, the re-expression of RAB39B in neurons or the re-establishment of AMPAR surface functionality by using the Ca^2+^-permeable AMPAR antagonist *N*-[3-[[4-[(3-aminopropyl)amino]butyl]amino]propyl]-1-naphthaleneacetamide-trihydrochloride (NASPM), but not GluA2 per se, causes the morphology and dynamic rate of *Rab39b* KO spines to revert.

Together, these results highlight a new role for AMPAR trafficking *via* RAB39B in spine remodelling, explaining how *RAB39B* loss-of-function mutations lead to the pathogenesis of XLID. We propose a new target suitable for future therapeutic interventions.

## Methods

### Animals

Experiments were performed in accordance with animal protocols approved by the “Institutional Animal Care and Use Committee (IACUC)” (IRCCS San Raffaele Scientific Institute, Milan, Italy) and by the Italian National Ministry of Health (IACUC ID 652, 653, 984), following the guidelines established by the European Community Council Directive D.L. 26/2014 on the use of animals in research (86/609/EEC). We minimised animal suffering, and we used only the number of animals necessary to produce reliable results. Animals were maintained on a 12 h light/darkness cycle, and an inverted cycle was employed in behavioural studies. Food pellets and water were available ad libitum unless stated otherwise.

### Generation of *Rab39b* knockout mice

The murine *Rab39b* gene mapped to the mouse X chromosome in XA7.3 occupying a region of 6187 base pairs (bp) (NC_000086.7). It is composed of two exons of 215 and 427 bp spaced out by one 2785 bp intron. The 5′- and 3′- untranslated regions (UTRs) flanking the two exons were 224 bp and 2536 bp, respectively.

**Fig. 1:**
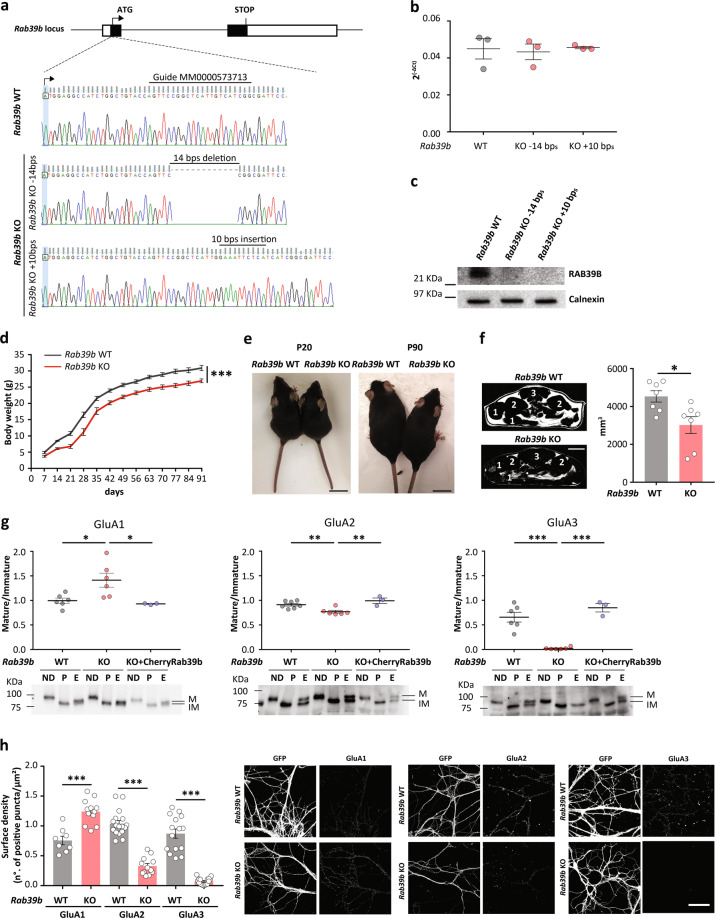
Generation of a *Rab39b* KO mouse model. **a** Scheme of the *Rab39b* locus: exons are black boxes spaced from one intron; 5′- and 3′-UTRs are white boxes. Enlargement of the *Rab39b* sequence to highlight the target sequence of the MM0000573713 guide (upper), 14 bp deletion (middle; −14 bp) and 10 bp insertion (bottom; +10 bp). **b** Expression profile of *Rab39b* transcripts in *Rab39b* WT, KO −14 bp and KO +10 bp brain lysates (*n* = 3). Data are expressed as *Rab39b* expression normalised to histone H3 (2^-ΔCt(Rab39b–H3)^). **c** Representative western blots showing RAB39B protein expression in *Rab39b* WT, KO −14 bp and KO +10 bp brain lysates (*n* = 3). Calnexin is the loading reference. **d** Body weight in grams (**g**) of *Rab39b* WT (*n* = 10) and KO (*n* = 11) littermate mice. **e** Representative images of 20- and 90-day-old *Rab39b* WT and KO littermate mice. The scale bar is 2 cm. **f** Representative MRI scan figures showing abdominal axial “pure fat” obtained by image subtraction (T1 weighted—T1 weighted with fat saturation); after subtraction, the signal of non-fat tissue becomes null. The following main anatomic landmarks were reported: 1-bowel, 2-kidneys, 3-spine and vertebral muscles. The scale bar is 0.5 cm. Graph indicates pure fat in mm^3^. **g** Quantification of the ratio between mature (M) and immature (IM) forms of AMPAR subunits after EndoHf digestion. Lower panels show representative western blots for GluA1 (WT = 6, KO = 6, KO + CherryRab39b = 3 independent lysates), GluA2 (WT = 7, KO = 6, KO + CherryRab39b = 3 independent lysates) and GluA3 (WT = 6, KO = 6, KO + CherryRab39b = 3 independent lysates) after PNGasef (P) or EndoHf (E) digestion; ND: nondigested neurons. **h** Quantification of the neuronal surface expression density of GluA1 (WT = 8, KO = 12 images), GluA2 (WT = 17, KO = 12 images) and GluA3 (WT = 15, KO = 16 images), expressed in the number of positive puncta/µm^2^ and representative images. The number of images belongs to a minimum of three experimental replicates. All the data are expressed as the mean ± SEM. The scale bar is 50 µm. *p < 0.05; **p < 0.01; ***p < 0.001.

*Rab39b*^*−/Y*^ mice (*Rab39b* KnockOut, KO) were generated by injecting three gRNAs (Sigma-Aldrich; #MM0000573711; #MM0000573712; #MM0000573713) targeting different DNA sequences in the 1st *Rab39b* exon, together with Cas9 mRNA in C57Bl/6N murine zygotes [24]. We obtained 15/76 pups carrying different insertion/deletion (*indel*) mutations, and we selected heterozygote females carrying a deletion of 14 bp or an insertion of 10 bp, giving a frame shift and a premature stop codon after 84 and 92 amino acids, respectively, from guide #MM0000573713. Possible off-targets were excluded by CCTop [25]. Heterozygous females were crossed with C57Bl/6N wild-type (WT) males (Charles River, Italy) to monitor the transmission of the mutant allele in the expected Mendelian segregation ratio of an X chromosome gene in N1 generation. Genotypes were determined by sequencing the portion of DNA surrounding the mutation (MyGATC from Eurofins Genomics; 1385-primer: 5′-TGTTTGTCACCCTGGCAGCATCG-3′) after PCR amplification with New_5′ARM_Sal_For (5′-GGTTGTCGACCAGGCCAGTGATGTTCTCGCGG-3′) and 1385 primers. Generations up to N6 were obtained by backcrossing *Rab39b* heterozygote females carrying a 14 bp deletion or a 10 bp insertion with C57Bl/6N WT males to establish murine lines. For all the experiments, except for those depicted in Fig. 1b, c, *Rab39b* KO 14-bp male mice and their WT littermates were used.

Because *RAB39B* is an X-linked gene and because *RAB39B*-related XLID disease is inherited as a recessive trait and is more common in male patients, we focused all experiments on male mouse littermates. For body weight, *Rab39b* WT and KO littermate mice were weighed every 7 days from 7 to 91 days of life (P). The brain, testes, kidney, liver, spleen, heart, lung, stomach of P20 and P90 *Rab39b* WT and KO littermate mice were weighed, and coordinates of brains were measured as described in [26].

### RNA isolation and qRT-PCR

Total RNA from hippocampal tissues was isolated with TRIzol reagent (Invitrogen, #15596018) according to the manufacturer’s instructions. 1 µg of total RNA was reverse transcribed with M-MLV (Invitrogen, #28025-013) following the manufacturer’s instructions to produce cDNA. Real-time PCR was performed with SYBR Green Universal Mix (Roche, #04707516001) on a Light Cycler 480 (Roche Diagnostics). Data quantification was performed via the comparative threshold cycle (*Ct*) method with formula 2^-(inputCt-controlCt)^, assuming that the efficiency was 2. *Rab39b* was amplified with mR39bF (5′-GGATACAGCGGGTCAAGAGAGG-3′) and mR39bR (5′-GTTGGTAATGTCAAATAAGAGAAGAC-3′) primers [1]. For normalisation, histone H3 was amplified with mHistone3F (5′-GTGAAGAAACCTCATCGTTACAGGCCTGGTAC-3′) and mHistone3R (5′-CTGCAAAGCACCAATAGCTGCACTCTGGAAGC-3′) primers [1].

### Metabolism-related parameters

To measure metabolism-related parameters, P90 *Rab39b* WT and KO littermate mice were housed individually.

#### Food and water intake

Food and water intake evaluation was performed by replacing sawdust with a sheet of 3 mm paper and 10 g food pellet in a Ø6 cm dish without a lid and giving mice a 15 ml tube full of water. We weighed the Ø6 cm dishes and 15 ml tubes with water to calculate the amount of food and water consumed. Food and water intake were measured during light and dark cycles for a total of 2 days. Food intake was measured as the difference in weight between the food put into the cage and that remaining at the end of the dark or light cycle, taking crumbs into account. Water intake was measured as the difference in weight between the 15 ml tube with water and that remaining at the end of the light or dark cycle.

#### Body temperature

Body temperature was measured by means of rectal thermometry with the TCAT-2AC Animal Temperature Control (Physitemp, New Jersey, USA) equipped with RET-3 Rectal Probe for Mice.

#### Glucose evaluation

The glycaemic index was measured by a Breeze2 monitoring system (Bayer, Leverkusen, Germany) at the basal level (mice fed ad libitum) and 12 h after food and water starvation.

[^18^F]-FDG was prepared for clinical use following the European Pharmacopeia, VIII edition. Fasting animals were injected with [^18^F]-FDG through the tail vein. 1 h after tracer injection, each animal was anaesthetised by mixing 2% isoflurane and 1% medical air; subsequently, animals were sacrificed. Blood samples were collected, and radioactivity was counted in a gamma counter (LKB Compugamma CS1282, Wallac). Radioactivity concentrations in tissue and blood due to radioactive decay were corrected (half-life of [^18^F] = 108.9 min) and expressed as % of the injected dose per gram of tissue.

#### Mouse magnetic resonance imaging

Magnetic resonance imaging was used to measure abdominal adipose tissue and was performed with a dedicated horizontal 7-Tesla scanner (Bruker, BioSpec 70/30 USR, Paravision 5.1, Germany) equipped with a gradient system characterised by an amplitude of 450/675 milliTesla/meter (mT/m), slew rate of 3400/4500 Tesla/meter/second (T/m/s) and a rise time of 140 milliseconds (ms), coupled with a dedicated volumetric mouse body coil. High-resolution T1-weighted sequences with and without fat saturation were acquired covering a body region from the hepatic dome to the inguinal canal, including the scrotum, in male mice. By image subtraction (T1 – T1 fat), a “pure fat” image stack was obtained on which adipose tissue volume was semiautomatically segmented and quantified with region growing function after clustering. Image processing and all the analyses were performed by using open source MIPAV software v.7.0.0 (and later versions) [27].

### Hippocampal and cortical cultured neurons, lentiviral transduction, plasmid transfection and NASPM treatment

Primary neuronal cultures were prepared as in [28] with minor modifications. Hippocampi or cortex were dissected from E18.5 embryos from *Rab39b* WT and KO females. After 15 min of incubation with 0.25% trypsin (Euroclone, #ECB3051D) in Hanks’ balanced salt solution (HBSS; LifeTech, #14170-088) at 37 °C, hippocampi or cortex were washed three times with HBSS to remove trypsin and then mechanically dissociated. Neurons were counted, and 150,000 cells were plated on poly-L-lysine (Sigma-Aldrich, #P2636; 0.2 mg/ml)-treated glass coverslips (Ø24 mm) for molecular biology experiments or 300,000 cells were plated on poly-L-lysine-treated plastic dishes (Ø3.5 cm) for biochemical experiments. Cells were plated in NM5 [neurobasal medium (LifeTech, #21103) supplemented with 5% foetal bovine serum (FBS; LifeTech, #16000-034), 2 mM glutamine (Life tech, #25030-024), 100 µg/ml PenStrep (LifeTech, #15140) and 2% B27 (LifeTech, #17504-044)]. After 2 h of incubation at 37 °C and 5% CO_2_ to allow adhesion to the substrate, the medium was changed to NM0 (NM5 without FBS).

Transduction was performed at MOI1 with lentiviral particles expressing GFP at 2 days in vitro (DIV) and CherryRab39b at 4 DIV as previously described [1, 13].

If transfected, 150,000 neurons/Ø24 mm coverslips were treated at 4 DIV with Lipofectamine 2000 (Invitrogen, #11668-027) following the manufacturer’s instructions with some modifications. In detail, conditioned NM0 medium was collected in a tube to be reused, and 1 ml of prewarmed neurobasal medium was added to the cells and incubated at 37 °C in a 5% CO_2_ humidified atmosphere. In the meantime, solution 1 and 2 were prepared. Solution 1 was prepared by mixing 1 µl Lipofectamine 2000 with 100 µl Optimem (Life Tech, #51985) and stored for 5 min at room temperature. Solution 2 consisted of 0.5 µg CherryGluA2 (a gift from Prof. Richard Huganir, Johns Hopkins University School of Medicine, Baltimore, Maryland 21205, USA) mixed in 100 µl Optimem. Solutions 1 and 2 were mixed, incubated for 30 min at 37 °C and then dispensed on cells. After 1 h of incubation, the neurobasal medium was replaced with new medium composed of half preconditioned NM0 and half fresh NM0.

If treated, 30 µM NASPM was added directly on 150,000 cells/Ø24 mm coverslips without changing the medium, twice a day (at 9 am and 5 pm) every day from 6 DIV to 13 DIV; then, live imaging was performed at 14 DIV.

### De-glycosylation assay

14 DIV-cultured *Rab39b* WT, *Rab39b* KO and *Rab39b* KO transduced with CherryRab39b [13] hippocampal neurons were lysed with Lysis Buffer [1% SDS, 2 mM EDTA, 10 mM HEPES pH 7,4, 10 mM sodium fluoride, 1 mM sodium orthovanadate, 1 mM β-glicerophosphate, 1x protease inhibitor cocktail (Sigma-Aldrich, #P8340)], boiled for 5 min and syringed eight times. Protein lysates were denatured for 15 min at 55 °C and digested with EndoHf (NEB, #P0703S) or PNGasef (NEB, #P0704S) for 2 h at 37 °C. SDS loading buffer (230 mM Tris-HCl pH 6.8, 6.85% SDS, 24% glycerol, 0.008% bromophenol blue, 5% DTT, 5% β-mercaptoethanol) was finally added to the samples. Samples were analysed by western blotting.

### Western blot

Mouse primary hippocampal neurons were lysed at 14 DIV in lysis buffer, boiled for 5 min and syringed eight times. Hippocampi from *Rab39b* WT and KO mouse brains at 30 days of age (P) were dissected and potterized ten times in homogenisation buffer [320 mM saccarose, 5 mM HEPES pH 7.4, 2 mM EDTA, 10 mM sodium fluoride, 1 mM sodium orthovanadate, 1 mM β-glycerophosphate, 1x protease inhibitor cocktail (Sigma-Aldrich, #P8340)], incubated for 10 min at +4 °C on rotation and then centrifuged at 13,500 g for 20 min at +4 °C.

SDS-PAGE was run with the appropriate percentage of polyacrylamide gel, and then western blots were performed by using appropriate primary and HRP-conjugated secondary antibodies (Supplementary Table 1). Protein bands were detected by a Chemidoc machine (Bio-Rad). ImageJ analysis software (NIH, Bethesda, MD, USA) with the ‘Analyse gels’ plugin was used to analyse western blots. The pixel size of each protein band was normalised to its relative housekeeping band; for phosphorylated proteins, the ratio between normalised phosphorylated and normalised total forms was also calculated.

### Golgi staining

Golgi staining was performed following the FD Rapid GolgiStain Kit (FD NeuroTechnologies, Columbia, MD, US). Pictures from neuronal apical dendrites in the CA1 region of the hippocampus were captured using an AxioImager: Zeiss AxioImager M2m equipped with a 100× objective [29]. Three mice per genotype and age were analysed, and five dendrites per hemisphere from one slice from each mouse were examined. The number of head spines was manually counted and normalised to a dendritic length of 5 µm measured by ImageJ software. The morphology of spines was described as the ratio of spine length and spine width as previously described [30]. A maturity ratio <2.5 represented mature mushroom-like spine morphology, a ratio between 2.5 and 6 indicated immature filopodia-like spines, and a ratio >6 denoted long spine protrusion.

### Immunofluorescence and confocal imaging

To visualise GFP and Cherry fluorescence, neurons were fixed for 15 min in fixing solution (4% paraformaldehyde (Sigma-Aldrich, #P6148) and 4% sucrose (Sigma-Aldrich, #S3089) in 120 mM sodium phosphate buffer, pH 7.4). Coverslips were rinsed three times with PBS and then incubated for 2 h at RT in a humidified chamber with goat serum dilution buffer (GSDB; 15% goat serum, 450 mM NaCl, 20 mM sodium phosphate buffer, pH 7.4) + 0.1% Triton X-100. Coverslips were then mounted with Vectashield (Vectalab, #H1200) after washing three times within 30 min with high salt buffer (HS: 500 mM NaCl, 20 mM sodium phosphate buffer, pH 7.4) and one time with 5 mM sodium phosphate buffer, pH 7.4.

To visualise GluA1, GluA2 and GluA3 exposed on the cell surface [13], cells were incubated for 5 min with primary antibodies targeting the N-terminal portion of GluA1, GluA2 and GluA3 subunits (Supplementary Table 1) diluted in neurobasal medium at 37 °C and in 5% CO_2._ Then, the cells were fixed in fixing solution and rinsed three times with PBS. Coverslips were incubated for 2 h at RT in a humidified chamber with the appropriate fluorophore-conjugated secondary antibody (Alexa Fluor, Invitrogen) diluted in GSDB without Triton X-100. Finally, coverslips were mounted with Vectashield (Vectalab, #H1200) after washing three times within 30 min with HS and one time with 5 mM sodium phosphate buffer, pH 7.4. Pictures of hippocampal neurons were captured using a Leica TCS SP8 SMD FLIM laser scanning confocal microscope equipped with an HC PL APO CS 2 63X (NA 1.4) oil-immersion objective. Multiple focal planes with z-spacing of 0.2 µm were deconvolved with the Deconvolution wizard of Huygens essential software (SVI, The Netherlands) by providing the *lif*-file and flattened by ImageJ maximum projection.

AMPAR subunits, relative to the area of GFP and/or Cherry signals, were measured by using the “Gran filter” plugin (circle sizes from 1 to infinity) of ImageJ.

To analyse spines, deconvolved and projected images were Gaussian blurred with ImageJ and imported in Volocity software (Quorum Technologies Inc, Ontario) to manually edit spines, defined as dendritic protuberances from the parent dendrite demarcated by a GFP-soluble signal. Edited spines were analysed by Cell Profiler software (Broad Institute, Cambridge, MA), and eccentricity, compactness, maximum ferret diameter values and skeletonised images were exported for each spine. Spine density was defined as head-spine/µm dendritic length. Each spine was manually categorised as either having a neck or not a neck. Spine morphology was categorised as follows: spines with necks were separated into thin and mushroom spines based on eccentricity (thin ≥ 0.8; mushroom < 0.8); spines without necks were divided into filopodia, stubby or blobby spines based on eccentricity, maximum ferret diameter, compactness and the presence of branches defined by the skeletonization process (filopodia: eccentricity > 0.8, maxferetdiameter > 2.5 µm; stubby spines were defined as eccentricity < 0.8 without branches; blobby spines were defined as compactness > 1 with branches). For presentation of confocal images, contrast was enhanced by linear methods by using ImageJ.

### Live imaging

Live imaging was performed with the samples in KRH solution (130 mM NaCl, 5 mM KCl, 1.2 mM KH_2_PO_4_, 1.2 mM MgSO_4_, 2 mM CaCl_2_, 25 mM HEPES, 6 mM glucose, pH 7.4, osmolarity 3.08–3.10). 200 frames every 10 s were recorded (~33 min) by a Leica SR GSD 3D TIRF equipped with an HC PL APO 160 X (NA 1.4 3) Oil Corr GSD TIRF Pifoc objective and iXon ultra 512 × 512 camera. Multiple focal planes (z-spacing 0.21 µm) were deconvolved with the Deconvolution wizard of Huygens essential software. Videos were aligned with the “Registration-Stackreg” plugin of FiJi and imported in Volocity to manually edit spines of 30 µm long dendrites and to determine the variation in pixel size of each spine in acquired frames. Values were imported in MATLAB, where spines were categorised as stable spines if present at all times and transient spines if they appeared and disappeared during track recording, and the dynamic rate of each spine was calculated as the pixel size variance of the spine area normalised by the area at time zero [31]. For image presentation, contrast was enhanced by the linear method by ImageJ.

### Electrophysiology

Coronal slices that were 200–250 μm thick and that contained the mPFC were prepared from P60 *Rab39b* WT and KO mice. Slices were kept for 30 min in artificial cerebrospinal fluid (aCSF) containing 119 mM NaCl, 2.5 mM KCl, 1.3 mM MgCl_2_, 2.5 mM CaCl_2_, 1.0 mM NaH_2_PO_4_, 26.2 mM NaHCO_3_ and 11 mM glucose that was bubbled with 95% O_2_ and 5% CO_2_ and then were transferred at room temperature in the same solution. All recordings were made from prelimbic cortex (PrL) layer III. The whole-cell current-clamp recording technique was used to measure spike probability. The pipette internal solution contained (in mM) 140 K-gluconate, 2 MgCl_2_, 5 KCl, 0.2 EGTA, 10 HEPES, 4 Na_2_ATP, 0.3 Na_3_GTP, 10 phosphocreatine, pH 7.3 and 300 mOsm. Action potentials (APs) were elicited by injecting small current steps of 50 pA starting at 0 and continuing to 500 pA. APs were counted for each step. To isolate mEPSCs, recordings were made in a voltage-clamp configuration, and neurons were held at −70 mV. 1 μM tetrodoxin and 50 μM picrotoxin (PTX) were added to the bath solution, and neuronal activity was recorded for 30 min (5 min baseline and 25 min of mEPSCs). mEPSCs were recorded with a Multiclamp 700B amplifier (Axon Instruments, Foster City, CA), filtered at 2.2 kHz, digitised at 5 Hz, and analysed using MiniAnalysis 6 (Synaptosoft) software.

Synaptic plasticity recordings were made in voltage-clamp configuration in aCSF + 50 μM PTX. Internal pipette solutions contained (in mM) 130 CsCl, 4 NaCl, 2 MgCl_2_, 1.1 EGTA, 5 HEPES, 2 Na_2_ATP, 5 sodium creatine phosphate, 0.6 Na_3_GTP, 0.1 spermine and 5 lidocaine N-ethyl bromide, pH 7.3; osmolarity was adjusted to 289 mOsm. EPSCs were elicited by placing a unipolar electrode onto PrL layer 5. Access resistance (10–30 MΩ) was monitored by a hyperpolarizing step of −4 mV at each sweep every 10 s. Data were excluded when the resistance changed >20%. The AMPA/NMDA ratio was calculated by subtracting the mixed AMPA-NMDA EPSC (+40 mV), the AMPA component isolated by D-APV (50 µM at +40 mV) bath application. The rectification index (RI) of AMPARs is the ratio of the chord conductance calculated at a negative potential (–60 mV) divided by the chord conductance calculated at a positive potential (+40 mV). For isolation of AMPA currents sensitive to NASPM, cells were clamped at −70 mV in aCSF + 50 μM PTX, and EPSCs were elicited. After 5 min (Pre), NASPM (15 μM) was added to the recording chamber, and the cells were recorded for 15 min (Post). The ratio of the Pre-Post response was calculated. The analysis of the decay time of NMDAR-mediated EPSCs was conducted as described previously [32], and ifenprodil sensitivity was calculated as the percentage of NMDAR-EPSC amplitude reduction (at +40 mV) after 30–40 min of continuous ifenprodil (3 μM, GluN2B-containing NMDAR antagonist) bath application compared to baseline. Synaptic responses were collected with a Multiclamp 700B amplifier (Axon Instruments, Foster City, CA), filtered at 2.2 kHz, digitised at 5 Hz, and analysed online using Igor Pro software (Wavemetrics, Lake Oswego, OR).

### Behavioural tests

All behavioural tests to compare *Rab39b* WT and KO littermate male mice were performed on 3- to 4-month-old mice. Animals were video-tracked with the EthoVision 15XT system (http://www.noldus.com) and analysed with Wintrack 2.4 (http://www.dpwolfer.ch/wintrack). For fear conditioning protocols, the ANY-maze system was used (https://www.anymaze.com).

#### Emergence and novelty test

Frames of nonreflective aluminium (37 cm high) were used to divide the maze into four arenas (50 × 50 cm), allowing concurrent observation of four mice.

For the emergence test, 24 h before testing, a home box (12 × 8 × 4 cm) with an opened door (8 × 4 cm) was inserted into the mouse’s home cage. The next day, the home box was placed 5 cm from the corner of the testing arena with the opened door facing the centre of the arena. Then, the mouse was released in the centre of the arena and tracked for 30 min.

For the novelty test, performed the day after the emergence test, the arena setup was the same as for the emergence test without the home box. The test consisted of two phases of 30 min; in the first phase, the mouse was able to explore the known arena freely, and during the second phase, an object (50 ml Falcon tube) was vertically glued in the centre of the arena.

Horizontal activity was analysed by segmenting the recorded tracks into three motion states: resting, scanning and progressing. To assess the approach-avoidance conflict towards the arena, three concentric zones were defined: exploration (55% of the arena in the emergence tests and 10% in the novelty tests), transition (29% of the arena in the emergence tests and 54% in the novelty tests) and home zones (16% of the arena in the emergence tests and 36% in the novelty tests) [33].

#### Water maze

Mice were trained in a circular pool of (Ø150 cm; 50 cm height) according to a standardised protocol [34]. In the hidden platform (14 × 14 cm) version of the water maze, mice had to locate the platform in a fixed position. The test included an acquisition phase (18 trials, 6 per day, intertrial time 30–40 min) followed by a reversal phase during which the platform was moved to the opposite position (12 trials, 6 per day). The first 60 s of trial 19 (the first reversal trial) was considered a probe trial. For the analysis, the trials were averaged in blocks of two trials.

#### Radial maze

The apparatus consisted of eight arms with a distance of 47 cm from a central platform. A cup with a food pellet was placed at the end of each arm. Food-deprived mice (maintained at 85% of their free-feeding weight) were placed in the centre platform and allowed to collect pellets placed at the end of each arm for 10 min. After 1 day of habituation to the maze, the mice were tested for 10 days. For each trial, the number of correct choices before the first error and the total number of errors were recorded [35].

#### Spontaneous alternation

A cross maze was obtained by closing 4 of the 8 arms of the radial maze apparatus. Each mouse was released in the centre platform and allowed to explore the maze for 10 min. The number and sequence of arm entries were recorded throughout the experiment. A correct alternation was considered when no more than one repetition over five entries was made. The percentage of correct spontaneous alternations and the total number of visits were calculated [36].

#### Fear conditioning

All mice were pre-exposed to the test chamber (Ugo Basile, Italy) for 10 min on the day preceding the training session (conditioning) to reduce the salience of context cues during conditioning. During the training session of the standard procedure, the trial started with a 1 min adaptation followed by the presentation of the tone (CS, 15 s) superimposed with a foot shock for the last 2 s (US). In the trace procedure, CS and US phases were separated by a trace (15 s). For both protocols, CS-US association was repeated five times with 60 s intertrial intervals (ITIs). The context test and tone test took place 24 h after conditioning. The context test consisted of 2 min without CS in the same training chamber, and the tone test consisted of 1 min without CS followed by 1 min with CS in a new environment. During each trial, the frequency of freezing (absence of movements except respiration) was continuously recorded [37].

### Statistical analysis

Graphs and statistical analysis were performed by GraphPad Prism v7 software, except for the analysis of the dynamic rate for which MatLab was used. The Shapiro–Wilk normality test was performed to assess the normal distribution of data. When normally distributed, the data were analysed with independent *t*-tests and repeated measures (RM) ANOVA as appropriate. If the data violated the normality test, the following nonparametric unpaired *t*-tests were performed: Kruskal–Wallis test followed by post hoc Dunn’s test or Mann–Whitney U test, as appropriate. Mood’s test was used to compare the medians of two samples. For the analysis of variance with two factors (two-way ANOVA, RM two-way ANOVA and RM two-way ANOVA by both factors), normality of sample distribution was assumed, followed by Bonferroni post hoc test.

*P* values < 0.05 were considered statistically significant and are detailed in Supplementary Table 2.

## Results

### *Rab39b* KO mice generation and characterisation

To analyse the pathophysiological contribution of the lack of RAB39B to XLID, *Rab39b* KO mice were generated by CRISPR/Cas9 technology [24] (Fig. 1a), and *Rab39b* WT and KO littermate male mice were evaluated. qRT-PCR and western blot analysis of hippocampal *Rab39b* WT and KO brain lysates showed intact *Rab39b* mRNA transcripts and loss of RAB39B protein expression in *Rab39b* KO mice compared to WT mice (Fig. 1b, c).

The evaluation of *Rab39b* KO mouse body weight, every 7 days from 7 to 91 days (P), showed a 10% body weight reduction compared to WT, starting in the early stage of development (P7) and that was maintained at each age analysed (*p* = 0.0001; Fig. 1d, e).

To understand whether the decrease in body weight involved the entire organism, all the organs were weighed in young (P20) and adult (P90) mice; no significant differences were observed between genotypes (Supplementary Fig. 1a). Recently, Zhang et al. described macrocephaly in *Rab39b* KO mice [38] that was not observed in a recent publication about a new mouse model [39]. Because macrocephaly is characterised by extreme overgrowth of cortical brain lengths [26], analysis of cortical measures at P20 and P90 in our *Rab39b* KO mice did not reveal any differences between genotypes, excluding the macrocephaly phenotype (Supplementary Fig. 1b, c).

To investigate the cause of the loss of body weight in *Rab39b* KO mice, we focused on adult mice (P90). We first monitored food and water intake and body temperature without finding any differences between genotypes (Supplementary Fig. 1d, e). Then, we determined possible variations in circulating blood glucose levels by measuring the glycaemic index (Supplementary Fig. 1g) and glucose consumption in the cortex, striatum, hippocampus, thalamus and cerebellum brain regions by using [^18^F]Fluoro-2-deoxy-d-glucose (FDG) tracer (Supplementary Fig. 1h); no differences were detected between P90 *Rab39b* WT and KO mice. By magnetic resonance imaging, we revealed that the loss of body weight in *Rab39b* KO mice could be ascribed to a significant reduction in the volume of abdominal adipose tissue (*p* = 0.015; Fig. 1f).

However, *Rab39b* KO male mice were viable and fertile, and the mutant allele was transmitted in the expected Mendelian segregation ratio of an X-linked gene.

### RAB39B governs GluA2/3 AMPAR trafficking

Because we previously reported that 80% RAB39B downregulation by shRNA impairs GluA2/GluA3 AMPAR and ER-to-Golgi trafficking, ultimately affecting the AMPAR composition at the neuronal surface [13], we investigated how the complete deletion of RAB39B impacts GluA1, GluA2, and GluA3 AMPAR subunit secretory pathways and their surface expression.

To analyse the ER-to-Golgi trafficking of AMPAR subunits in *Rab39b* KO primary hippocampal neurons, N-glycosylation status was analysed. AMPAR subunits are N-glycosylation proteins, and N-glycans from the ER to the Golgi complex are modified to be driven to the plasma membrane. *Rab39b* WT and KO primary hippocampal neurons were digested with Endo-b-N-acetylglucosaminidase H (EndoH), the enzyme that removes only unmodified N-glycans [40]. By immunoblotting, AMPAR subunits resulted in two isoforms after EndoH digestion, differing in their mobility in gel; these isoforms were an upper band, corresponding to the EndoH-resistant mature isoform, and a lower band, corresponding to the EndoH-sensitive immature isoform. The ratio between the two bands was a measure of subunit maturation (maturity ratio). As a control, neurons were digested with peptide-N-glycosidase F (PNGasef), which removes all N-glycans. We determined that GluA2 and the more striking GluA3 maturity ratios were significantly decreased in *Rab39b* KO neurons compared to WT neurons (GluA2 *p* = 0.004; GluA3 *p* < 0.0001; Fig. 1g), suggesting ER protein retention. In contrast, the GluA1 maturity ratio showed an increase (*p* = 0.02; Fig. 1g). Transducing *Rab39b* KO neurons with lentiviral particles expressing CherryRab39b did not affect the total amount of AMPAR subunits (Supplementary Fig. 1i), whereas GluA1, GluA2 and GluA3 maturity ratios were completely restored to those of WT conditions (*Rab39b* KO vs *Rab39b* KO + CherryRab39b; GluA1 *p* = 0.05, GluA2 *p* = 0.004, GluA3 *p* < 0.0001; Fig. 1g). Because alteration of the AMPAR subunit secretory pathway alters receptor surface expression [13], we next examined AMPAR surface density by immunolabelling *Rab39b* WT and KO hippocampal neurons lacking permeabilization. We showed decreased GluA2 and GluA3 surface density and increased GluA1 surface density on *Rab39b* KO neurons compared to the surface density observed on WT neurons (GluA1, GluA2 and GluA3 *p* < 0.0001; Fig. 1h).

Together, these data indicated that the complete lack of RAB39B specifically impairs GluA2/GluA3 heterotetrameric trafficking, regulating cell surface expression. Moreover, the increase in GluA1 maturation, not observed under downregulation conditions [13], with a concomitant increase in surface expression, suggests increased trafficking of homomeric GluA1 AMPAR.

### RAB39B is critical in spine development

**Fig. 2:**
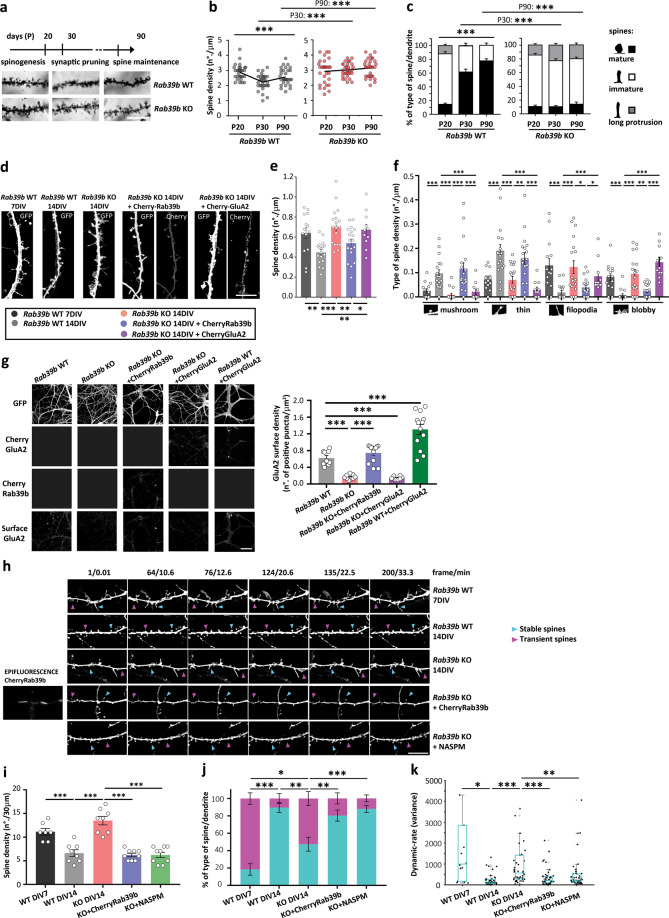
RAB39B-driven trafficking controls spine refinement. **a** Timeline of hippocampal dendritic spine development: spinogenesis at 20 days (P), synaptic pruning at P30 and spine maintenance at P90. Representative images of Golgi-impregnated CA1 hippocampal apical dendrites of *Rab39b* WT and KO mice (*n* = 3). The scale bar is 10 µm. **b** Spine density in number of spine heads/µm. **c** Partitioning of spine morphology along a dendrite in % of type of spine/dendrite. **d** Representative fluorescence images of *Rab39b* WT and KO primary hippocampal neurons fixed at 14 DIV and/or 7 DIV and *Rab39b* KO neurons transduced with CherryRab39b or transfected with CherryGluA2 (WT 7 DIV = 14; WT 14 DIV = 18; KO 14 DIV = 18; KO 14 DIV + CherryRab39b = 18; KO 14 DIV + CherryGluA2 = 11 dendrites from three independent preparations). The scale bar is 5 µm. **e** Spine density as the number of spine heads/µm. **f** Spine morphology as the type of spine/µm. **g** Representative images of primary hippocampal neuronal dendrites and quantification of the neuronal surface expression density of GluA2 as the number of positive puncta/µm^2^ of cultured GFP-transduced *Rab39b* WT (*n* = 9 images), KO (*n* = 9 images), KO transduced with CherryRab39b (*n* = 13 images) or transfected with CherryGluA2 (*n* = 8 images) and WT transfected with CherryGluA2 (*n* = 13 images). Images are from at least three independent experiments. The scale bar is 50 µm. **h** Representative live imaging frames of primary hippocampal neuronal dendrites of GFP-transduced *Rab39b* WT 7 DIV (*n* = 7) and 14 DIV (*n* = 8), KO 14 DIV (*n* = 7), KO transduced with CherryRab39b (*n* = 9) and KO after NASPM treatment (*n* = 9). *n* dendrites are from at least three independent experiments. Examples of spine behaviour: stable spines in cyan and transient spines in magenta arrows. The scale bar is 10 µm. **i** Spine density expressed as spine-head/30 µm dendritic length. **j** % of stable or transient spines/dendrite. **k** Dynamic rate as the pixel size variance within the stable spines. Data are the mean ± SEM except for (**k**), where the box-central mark is the median, the 25th and 75th percentiles are bottom and top edges, and ‘+’ are outliers. *p < 0.05, **p < 0.01, ***p < 0.001.

The coordination of GluA2-AMPAR subunit expression and targeting during neuronal development participates in synaptic network establishment through precise dendritic spine morphological reorganisation of excitatory neurons [41, 42], and alterations in AMPAR composition are generally observed in young animals; these alterations are associated with immature synapses and cognitive impairment [16, 43]. IDs together with a broad spectrum of neurodevelopmental disorders are characterised by defects in spine morphogenesis and/or refinement, often resulting in abnormal dendritic spine number and morphology [44]. We also determined whether the lack of RAB39B might influence dendritic spine development by analysing the spine density (Fig. 2a, b) and morphology (Fig. 2a, c) of apical dendrites of glutamatergic neurons in the CA1 area of the hippocampus of *Rab39b* WT and KO brains by Golgi staining. Analysis was performed at salient time points of hippocampal neuronal development at P20, P30 and P90, corresponding to spinogenesis [19], synaptic pruning [45] and spine maintenance [21], respectively (Fig. 2a).

In agreement with what was previously described [46], during development, *Rab39b* WT spines underwent a significant reorganisation of their density (P20 vs P30 vs P90: *p* < 0.0001; Fig. 2b) and their morphology (mature, immature, long protrusions *p* < 0.0001; Fig. 2c). The evaluation of *Rab39b* KO hippocampi revealed that no reorganisation in spine density and morphology occurred and that spines remained in a P20-like scenario (Fig. 2a–c).

Moreover, investigation of the genotype effect on the developmental time points did not show differences in *Rab39b* WT and KO neurons in spine density and morphology at P20 but differences were observed at P30 and P90 (density: P30 *p* < 0.0001, P90 *p* = 0.0003; morphology: mature, immature, long protrusions P30 *p* < 0.0001, mature, immature, long protrusions P90 *p* < 0.0001; Fig. 2b, c). These data suggest that RAB39B is not essential for the early stages of spine development, such as spinogenesis, but it is crucial during spine pruning and refinement periods.

To address the hypothesis that GluA2-AMPAR subunit RAB39B-mediated trafficking is required to guarantee spine reorganisation, we investigated the impact of RAB39B on spine pruning and remodelling. In primary hippocampal neurons, spine pruning and remodelling occur between 7 and 9 DIV, eliciting final neuronal maturation at 14 DIV [47], according to in vivo brain development (Fig. 2a) [21, 45]. We performed in vitro rescue experiments by re-expressing RAB39B or GluA2 at 4 DIV—before the gross morphological changes occurred between 7 and 9 DIV—to evaluate the in vitro long-term fate of *Rab39b* KO hippocampal spines at 14 DIV.

After validation of proper in vitro spine pruning and remodelling of WT spines comparing *Rab39b* WT spines between 7 and 14 DIV (density *p* = 0.0021; morphology: mushroom *p* = 0.0001, thin, filopodia, blobby *p* < 0.0001; Fig. 2d–f), we also confirmed an immature spine arrangement of *Rab39b* KO spines at 14 DIV that were comparable to WT at 7 DIV but were significantly different from WT at 14 DIV (density *p* < 0.0001; morphology: mushroom, thin, blobby *p* < 0.0001, filopodia *p* = 0.0003; Fig. 2d–f).

The expression of CherryRab39b or CherryGluA2 in *Rab39b* KO hippocampal neurons revealed that spine reorganisation was fully recovered by reintroducing RAB39B (density *p* = 0.008; morphology: mushroom *p* < 0.0001, thin *p* = 0.03, filopodia *p* = 0.02, blobby *p* = 0.002; Fig. 2d–f) but not GluA2 (Fig. 2d–f). In line with the predicted model, GluA2-AMPAR surface expression was restored only by reintroducing RAB39B in *Rab39b* KO hippocampal neurons but not GluA2 (*Rab39b* WT vs KO *p* < 0.0001; *Rab39b* KO vs KO + CherryRab39b *p* < 0.0001; *Rab39b* WT vs KO + CherryGluA2 *p* < 0.0001; Fig. 2g). As a control to exclude possible defects in CherryGluA2 plasmid expression, CherryGluA2 was expressed in WT neurons, showing an increased GluA2 surface density compared to WT neurons (*Rab39b* WT vs WT + CherryGluA2 *p* = 0.0002; Fig. 2g).

In conclusion, RAB39B-mediated vesicle trafficking is necessary and sufficient for spine elimination and remodelling towards a mature spine state.

### RAB39B deficiency leads to increased spine dynamics, rescued by acting on GluA2-lacking AMPARs

Having assessed the essential role of RAB39B-mediated trafficking in the orchestration of dendritic spine maturation, it was still not clear how RAB39B influences correct spine remodelling; does the absence of RAB39B freeze the spine in an immature state or does it increase the spine dynamic by not allowing it to stabilise itself in a mature state?

A live imaging technique was used to evaluate the spine dynamics of in vitro hippocampal neurons over time (Fig. 2h–k).

First, we determined how WT spines typically behave in vitro during development (at 7 and 14 DIV). A decrease in spine density (*p* = 0.0006; Fig. 2i) validated the previous results (Fig. 2e) other than an increase of 70% stable spines, and a concomitant decrease in transient spines from 7 DIV to 14 DIV was observed (stable, transient *p* = 0.0003; Fig. 2j). In addition, the dynamic rate of stable spines significantly decreased from 7 to 14 DIV (*p* = 0.014; Fig. 2k), indicating that WT spines moved from an immature hyperdynamic state at 7 DIV to a mature and more stable arrangement at 14 DIV.

Following the *Rab39b* KO spine dynamic, spine density significantly increased compared to that of WT at 14 DIV (*p* < 0.0001; Fig. 2i), as also shown in Fig. 2e; moreover, regarding WT spines, 50% were transient spines and 50% stable were spines corresponding to an intermediate state between 7 DIV and 14 DIV (KO 14 DIV vs WT 7 DIV: stable, transient *p* = 0.017; KO 14 DIV vs WT 14 DIV: stable, transient *p* = 0.0017; Fig. 2j). Given the above results that defined *Rab39b* KO 14 DIV spines as immature and comparable to WT spines at 7 DIV (Fig. 2d–f), we expected to find more correspondence of spine behaviour between *Rab39b* KO at 14 DIV and WT at 7 DIV. Detailing the *Rab39b* KO-stable spine dynamic behaviour, these spines exhibited a dynamic rate comparable to WT spines at 7 DIV, but not to WT spines at 14 DIV (*p* = 4.17E-07; Fig. 2k), endorsing the immature behaviour of *Rab39b* KO dendritic spines.

To assess the direct correlation between the role of RAB39B and spine stabilisation, we reintroduced RAB39B by transducing *Rab39b* KO hippocampal neurons at 4 DIV with CherryRab39b and then analysed them at 14 DIV. Spine density (KO 14 DIV vs KO + CherryRab39b *p* < 0.0001; Fig. 2i) and spine dynamics (KO 14 DIV vs KO + CherryRab39b, stable, transient *p* = 0.009; dynamic rate *p* = 8.49E−04; Fig. 2j, k) were rescued so that they were comparable to WT 14 DIV conditions.

**Fig. 3:**
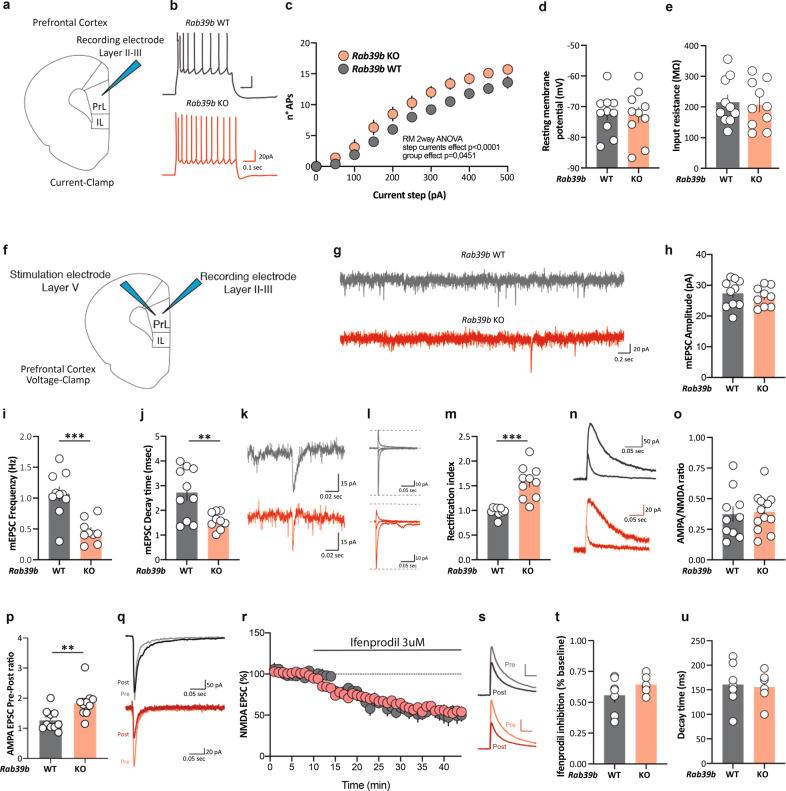
The lack of RAB39B impairs neuronal function. **a** Diagram of a patched region. **b** Example traces of pyramidal neuron excitability. **c** Summary data of the number of APs in response to 50 pA current steps. **d** Scatter plot of the resting membrane potential. **e** Scatter plot of the input resistance. **f** Diagram of a patched and stimulation region. **g** Example traces of mEPSCs. **h** Scatter plot of mEPSC amplitude. **i** Scatter plot of mEPSC frequency. **j** Scatter plot of mEPSC decay time. **k** Example traces of AMPA current decay time. **l** AMPAR-EPSCs example traces (−60, 0, and 40 mV). **m** Scatter plot of the rectification index recorded from cortical pyramidal neurons. **n** AMPAR/NMDA example traces at +40 mV. **o** Scatter plot of the AMPA/NMDA ratio recorded from cortical pyramidal neurons. **p** Scatter plot of AMPA currents sensitive to NASPM. **q** AMPA EPSC example traces before and after NASPM bath application. **r** Time course of NMDA EPSCs in the presence of ifenprodil (WT mice in grey, *Rab39b* KO mice in red). **s** NMDA EPSC example trace before and after ifenprodil bath application. **t** Scatter plot of ifenprodil inhibition calculated as % of baseline between 30 and 40 min after ifenprodil bath application. **u** Scatter plot of NMDA EPSC decay time before ifenprodil bath application. All the data are expressed as the mean ± SEM. *p < 0.05; **p < 0.01; ***p < 0.001. For each experiment 3 KO and 3 WT mice were used.

The nature of *Rab39b* KO dendritic spines may be ascribed to robust Ca^2+^ entry caused by increased Ca^2+^-permeable AMPAR surface expression (Figs. 1h and 3l) and by the absence of GluA2-RAB39B-mediated trafficking (Figs. 1g, h and 2g). *Rab39b* KO primary hippocampal neurons were treated with NASPM, a validated Ca^2+^-permeable AMPAR antagonist [48], between 6 DIV and 13 DIV, before spine stabilisation occurred. Live imaging at 14 DIV showed that NASPM treatment completely restored the dynamics of *Rab39b* KO spines so that they were comparable to the WT condition (KO 14 DIV vs KO + NASPM, stable, transient *p* < 0.0001; dynamic rate *p* = 0.001; Fig. 2h–j).

Our previous and present data thus far has demonstrated that the complete lack of RAB39B impairs GluA2/GluA3 trafficking, altering the availability of a correct AMPAR composition at the neuronal surface and determining neuronal Ca^2+^ permeability, which is directly responsible for dendritic spine turnover towards a mature synaptic state, as shown by RAB39B reintroduction in a KO environment or by NASPM treatment.

We also evaluated possible variations in the total amount of proteins involved in the direct modulation of spine formation, maturation and stabilisation [49–53], and no differences were detected between genotypes (Supplementary Fig. 2).

### The lack of RAB39B impairs neuronal function

It has been shown that during development, there is a switch in AMPA subunit composition. Indeed, cortical pyramidal neurons express GluA2-lacking AMPARs during early postnatal development when spinogenesis is at its maximum [54, 55]. We examined whether the absence of RAB39B could give rise to alterations in intrinsic electrical properties and excitatory synaptic transmission to cortical pyramidal neurons, that showed similar dendritic spine morphological alterations compared to WT (density *p* = 0.014; morphology: mushroom *p* = 0.0001, thin *p* = 0.0004, filopodia *p* = 0.034, blobby *p* < 0.0001; Supplementary Fig. 3) as *Rab39b* KO hippocampal neurons (Fig. 2d–f). Using a whole-cell patch clamp configuration, we first assessed the spike frequency of cortical pyramidal neurons in response to steps of current injections. As shown in Fig. 3b and c, *Rab39b* KO neurons had increased excitability compared to WT *Rab39b* neurons (*p* = 0.045) without a change in the resting membrane potential (Fig. 3d), or in the input resistance (Fig. 3e). We next performed recordings of AMPA miniature excitatory postsynaptic currents (AMPA-mEPSCs) in *Rab39b* WT and KO neurons. Under these conditions, we detected no differences in AMPA-mEPSC amplitude (Fig. 3h) but noted a decrease in the frequency (*p* = 0.0003; Fig. 3i) and decay time (*p* = 0.0062; Fig. 3j, k) in *Rab39b* KO neurons compared to WT neurons. We then evaluated whether the absence of RAB39B could alter synaptic strength in *Rab39b* KO neurons synapsing onto pyramidal cortical neurons by recording excitatory postsynaptic currents (EPSCs). We found an increase in the RI (*p* < 0.0001; Fig. 3l, m) and no changes in the AMPA/NMDA ratio (Fig. 3n, o). When we bath applied NASPM, a selective antagonist of Ca^2+^-permeable AMPA receptors, we found that pyramidal neurons recorded from KO mice showed a greater decrease in the AMPA amplitude than neurons recorded from WT mice, thus confirming the insertion of GluA2-lacking AMPA receptors (*p* = 0.0049, Fig. 3p, q). Subsequently, we tested ifenprodil sensitivity in pharmacologically isolated NMDA currents. We did not detect differences in ifenprodil inhibition (Fig. 3r–t) or in basal decay time kinetics (Fig. 3u), suggesting no alterations in NMDA receptor subunit composition. Together, these results suggest that the absence of RAB39B does not alter synaptic strength but increases the proportion of GluA2-lacking AMPARs in synapses, suggesting a lack of physiological switching of the AMPA receptor.

### *Rab39b* KO mice showed increased activity and impaired associative memory

To evaluate possible behavioural alterations comparable to those observed in ID features [1, 4], 3- to 4-month-old *Rab39b* WT mice and KO littermate adult male mice were subjected to a battery of behavioural tests to evaluate emotional and explorative behaviour and cognitive impairment.

**Fig. 4:**
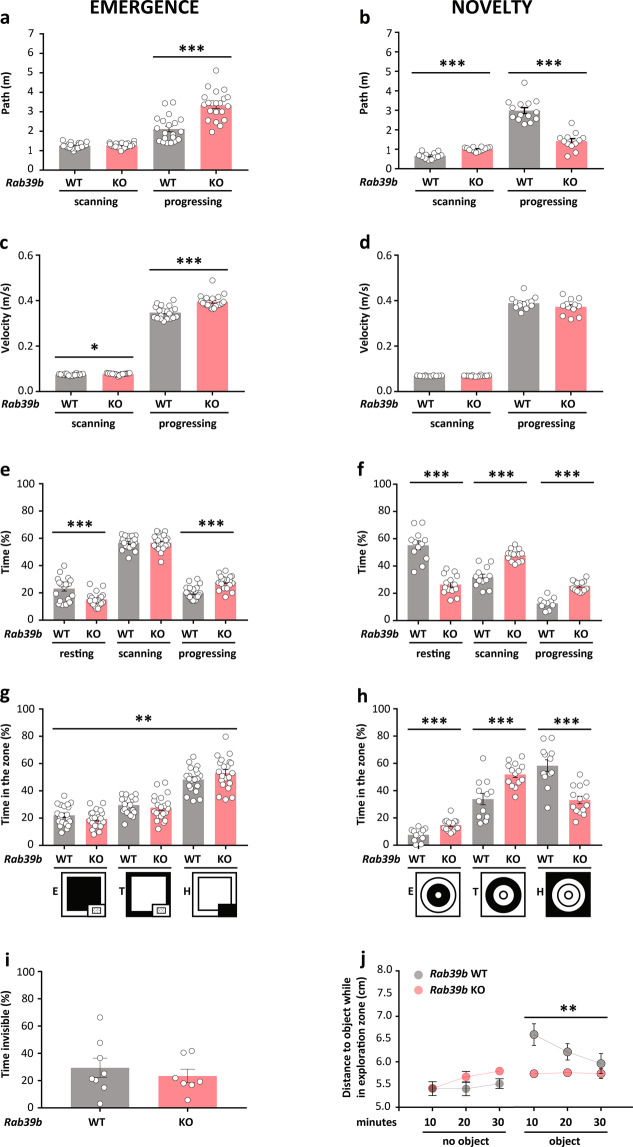
Increase activity and curiosity towards novelty in *Rab39b* KO. Comparison of (**a**, **c**, **e**, **g**, and **i**) emergence (WT = 20, KO = 21 mice) and (**b**, **d**, **f**, **h**, and **j**) novelty (WT = 12, KO = 14 mice) tests for several variables. **a**, **b** Distance travelled in metres. **c**, **d** Velocity in metres/second. **e**, **f** % of time spent in the three motion states: resting, scanning and progressing. **g**, **h** % of time in exploration (E), transition (T) and home (H) zones, highlighted in black in the pictograms below the graphs. **i** % of time spent in the home box (WT = 8, KO = 7). **j** Distance to the object while in the exploration zone in centimetres. Data are presented as the mean ± SEM. *p < 0.05; **p < 0.01; ***p < 0.001.

We examined explorative and emotional behaviour using emergence (Fig. 4a, c, e, g, i) and novelty (Fig. 4b, d, f, h, j) tests [33].

In the emergence test, locomotor activity, scored by total path and velocity, was significantly increased in *Rab39b* KO mice, as shown by a significant effect of path while progressing (*p* < 0.0001), for velocity during scanning (*p* = 0.013) and during progression (*p* < 0.0001) (Fig. 4a, c). These results were mirrored by a significant decrease at rest compared to the increase observed during progression (rest: *p* = 0.0006; progression: *p* < 0.0001; Fig. 4e).

In the novelty test, scanning activity was significantly increased in *Rab39b* KO mice, whereas progression was decreased (scan: *p* < 0.0001; progression: *p* < 0.0001; Fig. 4b) with no changes in velocity (Fig. 4d). Here, the absence of increased activity in *Rab39b* KO mice relied on the presence of an object in a yet-known arena, as shown by the significant difference observed in the percentage of time in rest compared to the increase observed during scanning and progression caused by the presence of an object (rest, scan, progression: *p* < 0.0001; Fig. 4f).

Mice approached and explored novel stimuli and the environment, but they avoided unfamiliar environments or objects by exhibiting fear-related responses. In the emergence test, the home box was a small familiar zone within a large unfamiliar arena, whereas in the novelty test, the novel stimulus was an object placed in the centre of an already familiar arena. Moreover, approach or avoidance in mice reflected the arena zone preferences of the mice (pictograms in Fig. 4g, h).

In the emergence test, *Rab39b* KO mice significantly preferred to stay in the less aversive home zone (*p* = 0.004; Fig. 4g) without hiding themselves in the home box compared to WT mice (Fig. 4i).

Contrastingly, in the novelty test, a significant difference between genotypes was observed in lack of avoidance of exploration (*p* = 0.0005) and transition (*p* = 0.0006) zones concomitant with a lower preference for the home zone (*p* < 0.0001; Fig. 4h). This phenotype of *Rab39b* KO mice was justified by an excess of object approaches without losing interest during the 30 min observation period (*p* = 0.010; Fig. 4j).

*Rab39b* KO mice had increased activity in the emergence test compared to control animals and curiosity towards novelty, as shown by increased object investigation.

We then examined learning and memory. In the water maze task [34], *Rab39b* KO mice were indistinguishable from WT mice, suggesting no deficit in spatial memory (Supplementary Fig. 4).

**Fig. 5:**
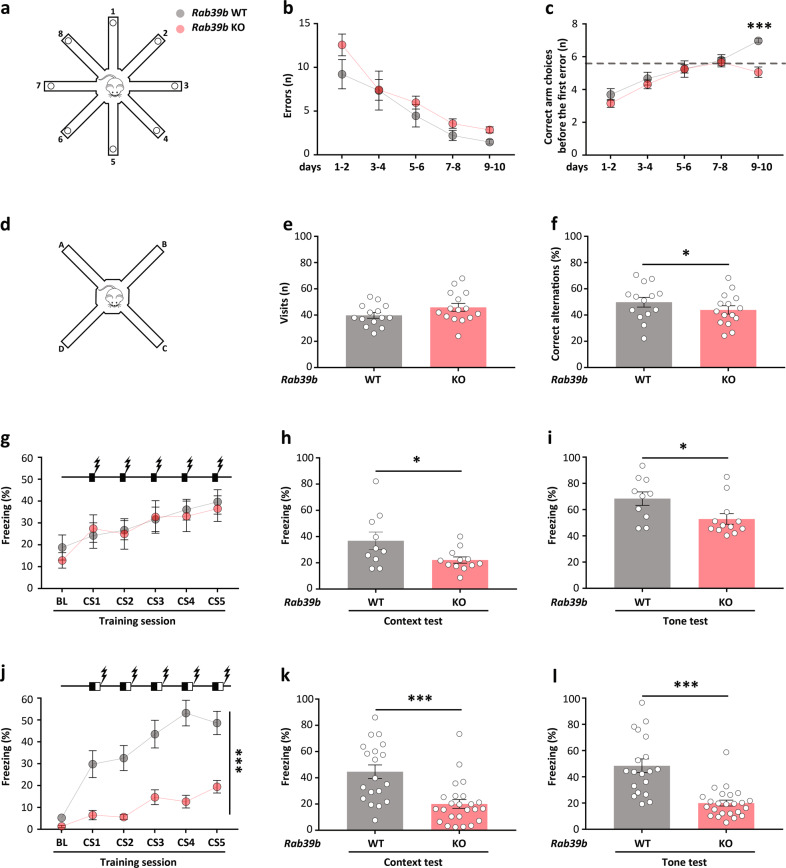
*Rab39b* KO mice are impaired in working and associative memory. **a** Pictogram of the 8-arm radial maze apparatus. Graphs indicate (**b**) the number or errors and (**c**) the number of correct arm choices before the first error during the 8-arm radial maze test (WT = 18, KO = 25 mice). The dashed line is the chance level performance of 5.5 correct successive arm visits. **d** Pictogram of the spontaneous alternation apparatus. Graphs show (**e**) the number of visits and (**f**) the % of correct alternations in the spontaneous alternation test (WT = 14, KO = 15 mice). **g**–**i** Standard and (**j**–**l**) trace fear conditioning protocols (standard: WT = 10, KO = 12; trace: WT = 19, KO = 24 mice). **g**, **j** % of freezing elicited by repeated exposure to the conditioned stimulus (CS) during the training session. BL indicates the baseline. Pictograms on the graphs represent (**g**) the standard fear conditioning protocol where the 15 s tone (CS, black box) is superimposed with a foot shock (lightning) for the last 2 s and (**j**) the trace fear conditioning protocol where the CS and the foot shock are separated by a 15 s trace (white box). (**h**, **i**, **k**, **l**) % of freezing during the memory test, 24 h after training, (**h**, **k**) for context, and (**i**, **l**) for tone. Data are presented as the mean ± SEM. *p < 0.05, ***p < 0.001.

In the eight-arm radial maze in which mice were to perform a spatial short-term working memory task (Fig. 5a–c), both *Rab39b* WT and KO mice showed a significant decline in the number of total errors over the 10 days of training, reflecting their ability to patrol the maze (WT: *p* < 0.0001; KO: *p* < 0.0001; Fig. 5b), and no difference was observed in the number of errors (Fig. 5b). *Rab39b* KO mice significantly differed from WT mice in correct responses (*p* = 0.05; Fig. 5c). Though WT mice had good performance (7 ± 0.18 out of 8 correct responses, Fig. 5c), KO mice did not exceed the chance level of 5.5 correct responses after 10 days of training (5 ± 0.32 out of 8 correct responses, *p* < 0.0001; Fig. 5c).

A similar deficit was observed in the spontaneous alternation task, a second working memory test (Fig. 5d–f), in that no differences were found in the number of visits (Fig. 5e) but the percentage of correct alternations was significantly different (*Rab39b* WT 54 ± 3% and KO 44 ± 3%; *p* = 0.04; Fig. 5f); this finding suggests that a lack of RAB39B impairs short-term working memory.

We finally assessed associative learning by auditory fear conditioning. In the training session of the standard procedure, no difference between genotypes in the percentage of freezing was detected (Fig. 5h), but during context and tone memory, *Rab39b* KO mice significantly differed from WT mice (context: *p* = 0.036; tone: *p* = 0.026; Fig. 5h, i). In trace fear conditioning, *Rab39b* KO mice froze significantly less than WT mice during conditioning (*p* < 0.0001; Fig. 5j) as well as in the subsequent two test sessions (context: *p* < 0.0001; tone: *p* < 0.0001; Fig. 5k, l). Both fear conditioning protocols revealed that the loss of RAB39B impairs associative memory.

## Discussion

As a follow-up to our previous work [13] and taking advantage of the *Rab39b* KO mouse model that we generated, we determined that the complete absence of RAB39B leads to a more excitable synaptic network and retained Ca^2+^-permeable AMPARs at synapses. This condition is directly associated with immature spines, which remain hyperdynamic and fail to prune at the postnatal stage. These altered neuronal circuits are mirrored in several behavioural traits, such as hyperactivity and memory deficits reminiscent of the human ID condition.

The remarkable inference from this study is that the aberrant expression of GluA2-lacking AMPARs in adult *Rab39b* KO synapses defined by the AMPAR maturation index, by AMPAR surface expression, and by the increase in the RI in *Rab39b* KO pyramidal cortical neurons as indicated by electrophysiological data at the functional level, is an important mechanism that regulates dendritic spine development. Indeed, this suggests that the canonical switch from GluA2-lacking to GluA2-containing AMPARs, characterising neuronal development [55], does not occur in *Rab39b* KO mice. GluA2-lacking receptors are typically Ca^2+^ permeable; thus, their activation induces an influx of calcium, which in turn could affect neuronal excitability. When directly injected with current, we found an increase in neuronal excitability in cortical pyramidal neurons of *Rab39b* KO mice compared to WT mice. This suggests that the absence of RAB39B could have a role in the regulation of neuronal intrinsic properties, and whether this is a direct or indirect effect needs to be further investigated. Moreover, though the absence of RAB39B does not alter either the synaptic strength or the NMDA subunit composition, we found that it induces alterations in AMPAR-mEPSC frequency and decay. Though the frequency is often associated with changes in the number of functional synapses, which can be ascribed to pre- or postsynaptic alterations, the decay time is linked to AMPA receptor composition; GluA2-containing AMPARs are faster, whereas GluA2-lacking AMPARs are slower [56, 57]. The altered receptor composition was confirmed by the reduction of AMPA amplitude following NASPM application. Our results support a redistribution of GluA2-lacking/GluA2-containing AMPARs in adult synapses of the cortex, which could be explained by the lack of a developmental switch between AMPAR subunits.

GluA2-AMPAR subunit switching correlates with synaptic network establishment through precise dendritic spine morphological reorganisation of excitatory neurons [41, 42]. One of the most prominent neuronal phenotypes observed in many neurodevelopmental disorders is the alteration in dendritic spine development—involving shape and/or number—suggesting that dendritic spines may serve as a common substrate for disorders that include deficits in cognition and information processing [44, 58]. The discrimination of where and when spine alterations occur in disease progression by analysing the time course of spine defects and their relationships with other endophenotypes in animal models may permit the identification of new therapeutic intervention opportunities. Here, we validated the persistence of spine aberrations in a *Rab39b* mouse model of ID in which RAB39B did not impair general spine development but specifically affected dendritic spine pruning and refinement. This “immaturity” phenomenon is widespread among different brain regions, as it has been shown that both the cortex and hippocampus are affected in the same direction by the lack of RAB39B.

Molecular networks controlling spines have been extensively studied; however, all the frameworks will be useful for understanding how a large number of rare genetic mutations can interrelate to disrupt spine morphology, synaptic function and neuronal circuit organisation, ultimately affecting behavioural performance in a disease-specific manner [59].

We demonstrated the direct cause-and-effect relationship between the increase in Ca^2+^-permeable AMPARs due to the absence of RAB39B and the phenotypic ID-like characteristic of spine morphogenesis alteration. Until now, studies have purely associated the negative effect of increased Ca^2+^-permeable AMPA receptors on dendritic spine morphogenesis [43, 60–62], and others have reported alterations in AMPAR surface expression in several animal models of ID [63–65] and in post-mortem brains from human patients [66].

Rescue experiments re-expressing RAB39B or GluA2 revealed that RAB39B trafficking is necessary and sufficient for spine elimination and remodelling towards a mature spine state. Moreover, modulating Ca^2+^ influx with NASPM [48] as well as re-expressing RAB39B, we were able to restore the high dynamic turnover of *Rab39b* KO spines to WT conditions. This indicates that the absence of RAB39B and the final amount of increased Ca^2+^-permeable AMPARs on synapses cause spine instability, in agreement with the described contribution of abnormal expression of GluA2-lacking Ca^2+^-permeable AMPARs in neurodevelopmental disorders [43, 67, 68]. Moreover, our result on the efficacy of NASPM on Ca^2+^-permeable AMPARs in recovering spine abnormalities assumes therapeutic value. NASPM is already under study in murine models for neurodevelopmental disorders [64, 68], even if it is not useful for human patients due to its inability to overpass the blood brain barrier.

Mechanistically, even if we did not detect any modifications in the total amount of proteins involved in the direct modulation of spine formation, maturation and stabilisation, we cannot exclude the involvement of Ca^2+^-dependent signalling pathways [69, 70]. Live imaging studies on protein trafficking and intracellular neuronal localisation should identify which proteins involved in spine maturation-related pathways are specifically affected by the lack of RAB39B.

We finally analysed the explorative and cognitive performance of *Rab39b* KO male mice to identify reminiscent ID patient phenotypes and to evaluate the impact of immature states of dendritic spines on behavioural performance. We found that *Rab39b* KO mice had increased locomotor activity in the emergence test that was not observed in the novelty test, possibly due to the presence of an object in a yet-known arena, as shown by the increased curiosity towards a novel object. This is in agreement with what was described in a similar *Rab39b*-null mouse [38] and contrary to what was observed in different genetically GluA2-modified mice without GluA2 subunits [71].

Concerning cognitive functions, *Rab39b* KO mice showed a slight deficit in the radial maze working memory task, which was also reported by Niu et al. with different learning paradigms [39] and a drastic impairment in trace fear conditioning.

Fear conditioning acquisition is characterised by a transient increase in neuronal surface expression of Ca^2+^-permeable AMPARs in the lateral amygdala and in the hippocampus; these AMPARs are subsequently converted in Ca^2+^-impermeable AMPARs for memory consolidation [72]. In line with this observation, we suggest that the cognitive deficit observed in *Rab39b* KO mice is caused by the complete absence of GluA2-AMPAR trafficking to the neuronal surface that hinders a Ca^2+^-permeable frozen AMPAR scenario.

Two recent studies [38, 39] reported the generation of *Rab39b* KO mice by using different techniques. Zang et al. [38] reported that *Rab39b* KO mice had cortical neurogenesis deficits, macrocephaly, and social and motor deficits. Similar behavioural performance was observed by Niu et al. [39], demonstrating short-term working memory deficits, but the authors did not observe macrocephaly in agreement with our results. As suggested [39], this discrepancy may be caused by defects in metabolic or different biological processes between different genetic backgrounds (C57Bl/6J vs C57Bl/6N) or by the mouse age analysed. Moreover, both studies identified abnormal activation of the PI3K-AKT-mTOR pathway, which is important in regulating cell differentiation, proliferation and metabolism during development. As we described previously, even if we did not detect any modifications in the total amount of proteins analysed, additional experiments that analyse this pathway in our model will be useful.

In summary, our study demonstrated the importance of RAB39B-mediated AMPAR trafficking in spine remodelling and in cognition and may contribute to explaining the aetiology of RAB39B-related ID.

## Supplementary information

Supplementary Figure Legends

Supplementary Fig 1

Supplementary Fig 2

Supplementary Figure 1.

Supplementary Figure 2

Supplementary Figure 3

Supplementary Figure 4
