# Supplementary material for: RAB39B-mediated trafficking of the GluA2-AMPAR subunit controls dendritic spine maturation and intellectual disability-related behaviour
Source: Mol Psychiatry. 2021 May 25;26(11):6531–49. doi: 10.1038/s41380-021-01155-5 (PMC8760075; doi:10.1038/s41380-021-01155-5)
Supplement: Supplementary file 1 — Supplementary Figure Legends [file 41380_2021_1155_MOESM1_ESM.docx]

**Supplementary Figure legend**

**Supplementary Figure 1. General characterization of *Rab39b* KO mice and the impact of RAB39B loss on total AMPAR subunit expression.** (a) Brain, testis, kidney, liver, spleen, heart, lung and stomach organ weights in grams of *Rab39b* WT and KO littermate mice at 20 (P20, WT=5, KO=9) and 90 (P90, WT=4, KO=8) days of life. (b, c) Brain coordinates in cm of *Rab39b* WT and KO littermate mice at (b) 20 days (P20, WT=5, KO=9) and (c) 90 days (P90, WT=8, KO=9) of life. (d, e) Food and water intake in grams in dark and light cycles (WT=12, KO=15). (f) Body temperature in °C (WT=9, KO=10). (g) Glycaemic index as mg of glucose per dl of blood at the basal level (bl) and after 12 hours of food/water starvation (WT=10, KO=9). (h) [^18^F]FDG brain tracer uptake in the cortex, striatum, hippocampus, thalamus and cerebellum expressed as % of injected dose/gram of tissue (WT=6, KO=7). (i) Representative western blots and quantiﬁcation of the total protein amount of GluA1, 2 and 3 AMPAR subunits in *Rab39b* WT, KO and KO+CherryRab39b primary hippocampal neuronal lysates (WT=6, KO=6, KO+CherryRab39b=3), expressed in relative pixel intensity to the housekeeping protein. Calnexin was used as the housekeeping protein. Anti-RAB39B antibody detects endogenous RAB39B in Rab39b WT neurons and flag-RAB39B in *Rab39b* KO neurons transduced with CherryRab39b, where the Cherry ORF is in opposite orientation compared to RAB39B, which is in turn tagged with FLAG as previously described [^13^](#_ENREF_13). Data are expressed as the mean±SEM.

**Supplementary Figure 2. Dendritic spine morphogenesis pathways are not affected by the absence of RAB39B.** (a) Quantiﬁcation of the total amount of the proteins described to be involved in pathways regulating synaptic pruning events including survival/apoptotic pathways, microglia-related pathways and the catenin/cadherin cell adhesion complex. Quantification is expressed in bands relative pixel intensity normalized to the housekeeping protein; for phosphorylated proteins, the ratio between phosphorylated and total forms is presented. Images show representative western blots of p70S6K T389 and the total p70S6K (n=3), S6Rp S235-236 and total forms (n=3), LC3-I and LC3-II (n=3), ERK1/2 T202-Y204 and total forms (n=3), CX3CL1 (n=3), CX3CR1 (n=5), CD200 (n=5), CD200R (n=3), CD47 (n=3), CD172α/Sirp-a (n=3), C1q (n=3), C3 (n=3), N-Catenin (n=3), and N-Cadherin (WT=4, KO=3). RAB39B is used as genotype loading control. Calnexin and vinculin are housekeeping proteins. (b) Quantification of the total amount of proteins involved in actin-dependent spine maturation and refinement, expressed as bands relative to pixel intensity normalized to housekeeping proteins. For phosphorylated proteins, the ratio between phosphorylated and total forms is shown. Images are representative western blots of Paxillin (n=3), β-PIX (n=3), GIT1 (n=3), PAK1/3 (n=3), ERC1 (n=3), RAC1 (n=4), ARP3 (WT=7, KO=6), Cortactin (n=3), β-actin (n=3), βIII-tubulin (n=3), myosinIIβ (n=3), CamKIIα T286-287 and total forms (n=3). RAB39B is used as genotype loading control (n=3). Calnexin and vinculin are housekeeping proteins. Data are presented as the mean±SEM.

**Supplementary Figure 3. *Rab39b* KO cortical neurons showed impaired spine density and morphology.** (a) Representative fluorescence images of GFP-transduced *Rab39b* WT and KO primary cortical neurons fixed at 14 DIV (WT=16; KO=13 dendrites from 3 independent preparations). The scale bar is 5 µm. (b) Spine density as the number of spine heads/µm. (c) Spine morphology as the types of spine/µm. Data are presented as the mean±SEM.

**Supplementary Figure 4. *Rab39b* KO mice** **show intact spatial memory**. Water maze task (WT=12, KO=14 mice). (a) Escape latency in seconds to locate the hidden platform position. (b) Percent of time spent in the zone opposite to the old goal (oo), left of the old goal (ol), in the old goal (og) and right of the old goal (or) target location during the probe trial. The dashed line represents the 12.5% chance level of swimming in that particular zone.

Data are presented as the mean±SEM.
