# Supplementary material for: RAB39B-mediated trafficking of the GluA2-AMPAR subunit controls dendritic spine maturation and intellectual disability-related behaviour
Source: Mol Psychiatry. 2021 May 25;26(11):6531–49. doi: 10.1038/s41380-021-01155-5 (PMC8760075; doi:10.1038/s41380-021-01155-5)
Supplement: Supplementary file 2 — Supplementary Fig 1 [file 41380_2021_1155_MOESM2_ESM.docx]

**Supplementary Table 1: List of primary antibodies used.**

| **Antibody against** | **Code number** | **Dilution** |
| --- | --- | --- |
| RAB39B | ^1^ | ^1^ WB 1:500 |
| GluA1 | Millipore, #MAB2263 | WB:1:1000; IF 1: 1:200 |
| GluA2 | Millipore; #MAB397 | WB:1:1000; IF 1: 1:200 |
| GluA3 | Alomone lab, #AGC-010 | WB:1:1000; IF 1: 1:100 |
| P70S6K T389 | Cell Signaling, #9205 | WB:1:1000 |
| P70S6K | Santa cruz, #sc230 | WB:1:3000 |
| S6Rp S235-6 | Cell Signaling, #2211 | WB:1:1000 |
| S6Rp | Cell Signaling, #2217S | WB:1:5000 |
| LC3 I-II | Novus, #NB100 | WB:1:1000 |
| ERK 1/2 Thr202 Tyr204 | Cell Signaling, #9101 | WB: 1:1000 |
| ERK 1/2 | Cell Signaling, #9102S | WB: 1:1000 |
| CX3CL1 | Abcam, #ab25088 | WB: 1:1000 |
| CX3CR1 | Abcam, #ab8020 | WB: 1:500 |
| CD200 | Proteintech, #14057-1-AP | WB: 1:1000 |
| CD200R | Thermo, #PA5-18952 | WB: 1:500 |
| TREM2 | R&D, #AF1729 | WB: 1:1000 |
| CD47 | Santa cruz, #sc12730 | WB: 1:500 |
| CD172a/Sirpα | R&D, #AF7307 | WB: 1:1000 |
| βIII-tubulin | SySy, #302302 | WB: 1:50 000 |
| myosinIIβ | Cell Signaling, #3404 | WB: 1:1000 |
| CamKIIα T286-7 | Cayman, #10011438 | WB: 1:500 |
| CamKIIα | Cayman, #10011437 | WB: 1:5000 |
| ARP3 | Cell Signaling, #4738T | WB:1:2000 |
| β-Actin | Sigma-Aldrich, #5441 | WB: 1:10 000 |
| N-Catenin | Santa cruz, #sc1498 | WB 1:1000 |
| N-Cadherin | BD, #610920 | WB 1:1000 |
| Paxillin | From I. de Curtis, Vita-Salute San Raffaele University, Milano, IT | ^26^ |
| β-PIX | From I. de Curtis, Vita-Salute San Raffaele University, Milano, IT | ^26^ |
| GIT1 | From I. de Curtis, Vita-Salute San Raffaele University, Milano, IT | ^26^ |
| PAK1/3 | From I. de Curtis, Vita-Salute San Raffaele University, Milano, IT | ^26^ |
| ERC1 | From I. de Curtis, Vita-Salute San Raffaele University, Milano, IT | ^26^ |
| Cortactin | From I. de Curtis, Vita-Salute San Raffaele University, Milano, IT | ^26^ |
