# Supplementary material for: RAB39B-mediated trafficking of the GluA2-AMPAR subunit controls dendritic spine maturation and intellectual disability-related behaviour
Source: Mol Psychiatry. 2021 May 25;26(11):6531–49. doi: 10.1038/s41380-021-01155-5 (PMC8760075; doi:10.1038/s41380-021-01155-5)
Supplement: Supplementary file 3 — Supplementary Fig 2 [file 41380_2021_1155_MOESM3_ESM.docx]

**Supplementary Table 2: Table of statistics**

| **Figure** | | **Comparison** | **Statistic** |
| --- | --- | --- | --- |
| **1d** | Body weight | genotype effect * time | Repeated Measures ANOVA F[1,19]=23, p=0.0001 |
| **1f** | MRI | Genotype effect | Student’s t-test p=0.015 |
| **1g** | Maturity ratio | GluA1, WT vs KO  GluA1, KO vs KO+CherryRab39b | Student’s t-test p=0.020  Student’s t-test p=0.050 |
| **1g** | Maturity ratio | GluA2, WT vs KO  GluA2, KO vs KO+CherryRab39b | Student’s t-test p=0.004  Student’s t-test p=0.004 |
| **1g** | Maturity ratio | GluA3, WT vs KO  GluA3, KO vs KO+CherryRab39b | Student’s t-test p<0.0001  Student’s t-test p<0.0001 |
| **1h** | Surface expression | GluA1, genotype effect | Student’s t-test p<0.0001 |
| **1h** | Surface expression | GluA2, genotype effect | Student’s t-test p<0.0001 |
| **1h** | Surface expression | GluA3, genotype effect | Student’s t-test p<0.0001 |
| **2b** | Golgi staining, Spine density | WT, time effect | Kruskal Wallis p<0.0001;  post-doc Dunn’s test: P20 *vs* P30 p<0.0001, P30 *vs* P90 p=0.04 |
| **2c** | Golgi staining, Spine morphology | WT mushroom-like mature spines, time effect | Kruskal Wallis p<0.0001;  post-doc Dunn’s test: P20 *vs* P30 p<0.0001, P30 *vs* P90 p=0.03 |
| **2c** | Golgi staining, Spine morphology | WT filopodia-like immature spines, time effect | Kruskal Wallis p<0.0001;  post-doc Dunn’s test: P20 *vs* P30 p<0.0001, P30 *vs* P90 p=0.02 |
| **2c** | Golgi staining, Spine morphology | WT long-protrusions, time effect | Kruskal Wallis p<0.0001;  post-doc Dunn’s test: long protrusions: P20 *vs* P30 p<0.0001 |
| **2b** | Golgi staining, spine density | P30, genotype effect | Mann Whitney U test p<0.0001 |
| **2b** | Golgi staining, spine density | P90, genotype effect | Mann Whitney U test p=0.0003; |
| **2c** | Golgi staining, spine morphology | P30 mushroom-like mature spines, genotype effect | Mann Whitney U test p<0.0001 |
| **2c** | Golgi staining, spine morphology | P30 filopodia-like immature spines, genotype effect | Mann Whitney U test p<0.0001 |
| **2c** | Golgi staining, spine morphology | P30 long protrusions, genotype effect | Mann Whitney U test p<0.0001 |
| **2c** | Golgi staining, spine morphology | P90 mushroom-like mature spines, genotype effect | Mann Whitney U test p<0.0001 |
| **2c** | Golgi staining, spine morphology | P90 filopodia-like immature spines, genotype effect | Mann Whitney U test p<0.0001 |
| **2c** | Golgi staining, spine morphology | P90 long protrusions, genotype effect | Mann Whitney U test p<0.0001 |
| **2e** | Immunofluorescence, spine density | *Rab39b* WT 7DIV *vs* 14DIV | Student’s t-test p=0.0021 |
| **2e** | Immunofluorescence, spine density | *Rab39b* KO 14DIV *vs* *Rab39b* WT 14DIV | Student’s t-test p<0.0001 |
| **2e** | Immunofluorescence, spine density | *Rab39b* KO 14DIV *vs* *Rab39b* KO+CherryRab39b | Student’s t-test p=0.008 |
| **2e** | Immunofluorescence, spine density | *Rab39b* WT 14DIV *vs* *Rab39b* KO+CherryGluA2 | Student’s t-test p=0.0003 |
| **2f** | Immunofluorescence, spine morphology | Mushroom, *Rab39b* WT 7DIV *vs* 14DIV | Mann Whitney U test p=0.0001 |
| **2f** | Immunofluorescence, spine morphology | Thin, *Rab39b* WT 7DIV *vs* 14DIV | Mann Whitney U test p<0.0001 |
| **2f** | Immunofluorescence, spine morphology | Filopodia, *Rab39b* WT 7DIV *vs* 14DIV | Mann Whitney U test p<0.0001 |
| **2f** | Immunofluorescence, spine morphology | Blobby, *Rab39b* WT 7DIV *vs* 14DIV | Mann Whitney U test p<0.0001 |
| **2f** | Immunofluorescence, spine morphology | Mushroom, *Rab39b* KO 14DIV *vs* *Rab39b* WT 14DIV | Mann Whitney U test p<0.0001 |
| **2f** | Immunofluorescence, spine morphology | Thin, *Rab39b* KO 14DIV *vs* *Rab39b* WT 14DIV | Mann Whitney U test p<0.0001 |
| **2f** | Immunofluorescence, spine morphology | Filopodia, *Rab39b* KO 14DIV *vs* *Rab39b* WT 14DIV | Mann Whitney U test p=0.0003 |
| **2f** | Immunofluorescence, spine morphology | Blobby, *Rab39b* KO 14DIV *vs* *Rab39b* WT 14DIV | Mann Whitney U test p<0.0001 |
| **2f** | Immunofluorescence, spine morphology | Mushroom, *Rab39b* KO 14DIV *vs* *Rab39b* KO+CherryRab39b | Mann Whitney U test p<0.0001 |
| **2f** | Immunofluorescence, spine morphology | Thin, *Rab39b* KO 14DIV *vs* *Rab39b* KO+CherryRab39b | Mann Whitney U test p=0.03 |
| **2f** | Immunofluorescence, spine morphology | Filopodia, *Rab39b* KO 14DIV *vs* *Rab39b* KO+CherryRab39b | Mann Whitney U test p=0.02 |
| **2f** | Immunofluorescence, spine morphology | Blobby, *Rab39b* KO 14DIV *vs* *Rab39b* KO+CherryRab39b | Mann Whitney U test p=0.002 |
| **2f** | Immunofluorescence, spine morphology | Mushroom, *Rab39b* WT 14DIV *vs* *Rab39b* KO+CherryGluA2 | Mann Whitney U test p=0.0002 |
| **2f** | Immunofluorescence, spine morphology | Thin, *Rab39b* WT 14DIV *vs* *Rab39b* KO+CherryGluA2 | Mann Whitney U test p<0.0001 |
| **2f** | Immunofluorescence, spine morphology | Filopodia, *Rab39b* WT 14DIV *vs* *Rab39b* KO+CherryGluA2 | Mann Whitney U test p<0.0001 |
| **2f** | Immunofluorescence, spine morphology | Blobby, *Rab39b* WT 14DIV *vs* *Rab39b* KO+CherryGluA2 | Mann Whitney U test p<0.0001 |
| **2g** | GluA2 surface density | *Rab39b* WT *vs* *Rab39b* KO | Student’s t-test p<0.0001 |
| **2g** | GluA2 surface density | *Rab39b* WT *vs* *Rab39b* KO+CherryRab39b | Student’s t-test p<0.0001 |
| **2g** | GluA2 surface density | *Rab39b* WT *vs* *Rab39b* KO+CherryGluA2 | Student’s t-test p<0.0001 |
| **2g** | GluA2 surface density | *Rab39b* WT *vs* *Rab39b* WT+CherryGluA2 | Student’s t-test p=0.0002 |
| **2i** | Spine dynamic,  Spine density | *Rab39b* WT 7DIV *vs* WT 14DIV | Student’s t-test p=0.0006 |
| **2i** | Spine dynamic,  Spine density | *Rab39b* KO 14DIV *vs* KO 14DIV | Student’s t-test p<0.0001 |
| **2i** | Spine dynamic,  Spine density | *Rab39b* KO 14DIV *vs* KO+CherryRab39b | Student’s t-test p<0.0001 |
| **2i** | Spine dynamic,  Spine density | *Rab39b* KO 14DIV *vs* KO+NASPM | Student’s t-test p<0.0001 |
| **2j** | Spine dynamic, type of spine | Stable and Transient spines, *Rab39b* WT 7DIV *vs* *Rab39b* WT 14DIV | Mann Whitney U test p=0.0003 |
| **2j** | Spine dynamic, type of spine | Stable and Transient spines, *Rab39b* KO 14DIV *vs* WT 7DIV | Mann Whitney U test p=0.017 |
| **2j** | Spine dynamic, type of spine | Stable and Transient spines, *Rab39b* WT 14DIV *vs* KO 14DIV | Mann Whitney U test p=0.002 |
| **2j** | Spine dynamic, type of spine | Stable and Transient spines, *Rab39b* KO 14DIV *vs* KO+CherryRab39b | Mann Whitney U test p=0.009 |
| **2j** | Spine dynamic, type of spine | Stable and Transient spines, *Rab39b* KO 14DIV *vs* KO+NASPM | Mann Whitney U test p<0.0001 |
| **2k** | Dynamic rate | *Rab39b* WT 7DIV *vs* WT 14DIV | Mood’s test p=0.014 |
| **2k** | Dynamic rate | *Rab39b* KO 14DIV *vs* KO 14DIV | Mood’s test p=4.147E-07 |
| **2k** | Dynamic rate | *Rab39b* KO 14DIV *vs* KO+CherryRab39b | Mood’s test p=8.49E-04 |
| **2k** | Dynamic rate | *Rab39b* KO 14DIV *vs* KO+NASPM | Mood’s test p=0.001 |
| **3c** | Action potential | *Rab39b* WT vs KO | Repeated measure 2-way ANOVA, current injection main effect F(2.205,39.69)=269.4, p <0.001, genotype main effect F(1, 18)=4.636, p=0.0451, current injection-genotype effect F(10,180)=1.908, p=0.0467 |
| **3i** | mEPSC frequency | Genotype effect | Unpaired t-test t=4.479, df=17, p<0.001 |
| **3j** | mEPSC decay time | Genotype effect | Unpaired t-test t=3.120, df=17, p=0.0062 |
| **3m** | Rectification index | Genotype effect | Mann-Whitney U test, p<0.001 |
| **3p** | AMPA currents sensitive to NASPM | Genotype effect | Mann Whitney test, Mann-Whitney U=014, p=0.0049 |
| **4a** | Emergence, path | genotype*motion state | two-way repeated measures ANOVA F[1,1]=32.23, p<0.0001 |
| **4a** | Emergence, path | genotype effect for path while progressing | one-way factorial analysis ANOVA F[1,39]=31.1, p<0.0001 |
| **4c** | Emergence, velocity | genotype*motion state | two-way repeated measures ANOVA F[1,1]=28.12, p<0.0001 |
| **4c** | Emergence, velocity | genotype effect for velocity while scanning | one-way factorial analysis ANOVA F[1,39]=6.76, p=0.013 |
| **4c** | Emergence, velocity | genotype effect for velocity while progressing | one-way factorial analysis ANOVA F[1,39]=29.84, p<0.0001 |
| **4e** | Emergence, time | genotype*motion state | two-way repeated measures ANOVA F[2,1]=13.6, p<0.0001 |
| **4e** | Emergence, time | genotype effect for time while resting | one-way factorial analysis ANOVA F[1,39]=14.09, p=0.0006 |
| **4e** | Emergence, time | genotype effect for time while progressing | one-way factorial analysis ANOVA F[1,39]=27.38, p<0.0001 |
| **4b** | Novelty, path | genotype*motion state | two-way repeated measures ANOVA F[1,1]=50.29, p<0.0001 |
| **4b** | Novelty, path | genotype effect for path while scanning | one-way factorial analysis ANOVA F[1,24]=41.36, p<0.0001 |
| **4b** | Novelty, path | genotype effect for path while progressing | one-way factorial analysis ANOVA F[1,24]=59.6, p<0.0001 |
| **4f** | Novelty, time | genotype*motion state | two-way repeated measures ANOVA F[2,1]=59.9, p<0.0001 |
| **4f** | Novelty, time | genotype effect for time while resting | one-way factorial analysis ANOVA F[1,24]=63.1, p<0.0001 |
| **4f** | Novelty, time | genotype effect for time while scanning | one-way factorial analysis ANOVA F[1,24]=46.6, p<0.0001 |
| **4f** | Novelty, time | genotype effect for time while progressing | one-way factorial analysis ANOVA F[1,24]=73.8, p<0.0001 |
| **4g** | Emergence, time | genotype effect for time in zones | two-way repeated measures ANOVA F[1,39]=9.34, p=0.004 |
| **4h** | Novelty, time | genotype*time in zones | two-way repeated measures ANOVA F[2,1]=20.78, p<0.0001 |
| **4h** | Novelty, time | genotype effect for time in exploration zone | one-way factorial analysis ANOVA F[1,24]=16.39, p=0.0005 |
| **4h** | Novelty, time | genotype effect for time in transition zone | one-way factorial analysis ANOVA F[1,24]=15.74, p=0.0006 |
| **4h** | Novelty, time | genotype effect for time in home zone | one-way factorial analysis ANOVA F[1,24]=25.57, p<0.0001 |
| **4j** | Novelty, distance to object | genotype effect for distance to object when in the exploration zone | two-way repeated measures ANOVA F[1,21]=6.84, p=0.010 |
| **5b** | Radial maze,  errors | WT, total errors over the 10 days of training | two-way repeated measures ANOVA F[17,4]=9.91, p<0.0001 |
| **5b** | Radial maze,  errors | KO, total errors over the 10 days of training | two-way repeated measures ANOVA F[24,4]=26.91, p<0.0001 |
| **5b** | Radial maze,  Correct choices | genotype effect | two-way repeated measures ANOVA F[1,41]=3.9, p=0.05 |
| **5b** | Radial maze,  correct choices | genotype effect for day 9-10 | two-way repeated measures ANOVA F[1,41]=22.28, p<0.0001 |
| **5f** | Spontaneus alternation, correct alternations | genotype effect | one-way factorial analysis ANOVA F[1,25]=4.7, p=0.04 |
| **5h** | Fear conditioning,  context | genotype effect | one-way factorial analysis ANOVA F[1,20]=5.0, p=0.036 |
| **5i** | Fear conditioning,  tone | genotype effect | one-way factorial analysis ANOVA F[1,20]=5.78, p=0.026 |
| **5j** | Trace fear conditioning,  training | genotype effect | two-way repeated measures ANOVA F[1,41]=44.45, p<0.0001 |
| **5k** | Trace fear conditioning,  context | genotype effect | one-way factorial analysis ANOVA F[1,45]=22.32, p<0.0001 |
| **5l** | Trace fear conditioning,  tone | genotype effect | one-way factorial analysis ANOVA F[1,45]=25.66, p<0.0001 |
| **S 3b** | Immunofluorescence, spine density | genotype effect | Student’s t-test p=0.014 |
| **S 3c** | Immunofluorescence, spine morphology | Mushroom, genotype effect | Mann Whitney U test p=0.0001 |
| **S 3c** | Immunofluorescence, spine morphology | Thin, genotype effect | Mann Whitney U test p=0.0004 |
| **S 3c** | Immunofluorescence, spine morphology | Filopodia, genotype effect | Mann Whitney U test p=0.034 |
| **S 3c** | Immunofluorescence, spine morphology | Blobby, genotype effect | Mann Whitney U test p<0.0001 |
